# Biological Applications of Organic Electrochemical Transistors: Electrochemical Biosensors and Electrophysiology Recording

**DOI:** 10.3389/fchem.2019.00313

**Published:** 2019-05-07

**Authors:** Liming Bai, Cristina García Elósegui, Weiqi Li, Ping Yu, Junjie Fei, Lanqun Mao

**Affiliations:** ^1^Key Laboratory of Environmentally Friendly Chemistry and Applications of Ministry of Education, College of Chemistry, Xiangtan University, Xiangtan, China; ^2^Beijing National Laboratory for Molecular Sciences, Key Laboratory of Analytical Chemistry for Living Biosystems, Institute of Chemistry, The Chinese Academy of Sciences, Beijing, China; ^3^School of Chemical Sciences, University of Chinese Academy of Sciences, Beijing, China

**Keywords:** organic electrochemical transistors, biosensing, electrochemical sensing, electrophysiology recording, conducting polymer

## Abstract

Organic electrochemical transistors (OECTs) are recently developed high-efficient transducers not only for electrochemical biosensor but also for cell electrophysiological recording due to the separation of gate electrode from the transistor device. The efficient integration of OECTs with electrochemical gate electrode makes the as-prepared sensors with improved performance, such as sensitivity, limit of detection, and selectivity. We herein reviewed the recent progress of OECTs-based biosensors and cell electrophysiology recording, mainly focusing on the principle and chemical design of gate electrode and the channel. First, the configuration, work principle, semiconductor of OECT are briefly introduced. Then different kinds of sensing modes are reviewed, especially for the biosensing and electrophysiological recording. Finally, the challenges and opportunities of this research field are discussed.

## Introduction

Transistors were invented by William Shockley and his coagent in 1947 for the precluded amplification efficiency and reliability of vacuum tubes. With the discovery of conducting conjugated polymers in the late 1970s, organic conducting polymer-based transistors have been widely investigated. Among of them, organic thin-film transistors have attracted much attention due to their broad range of applications, especially in biological system, which includes two types of transistors, i.e., organic field effect transistors (OFETs) and organic electrochemical transistors (OECTs). OFETs always use small organic molecules and organic conjugated macromolecules as semiconductor, in which the gate voltage is applied across the gate insulator and through field effect doping the gate electrode modulate the channel current. Differently, OECTs use the electrolyte medium between the channel and the gate electrode rather an insulator layer. When applying gate voltages, electrochemical doping or de-doping from the electrolyte modulate the channel current. In this case, the OECTs is easier to be used in biological system since most of biological reaction or species are occurred in an electrolyte medium. More importantly, compared with other kinds of OFETs, OECTs always bear low working voltages (below 1 V), essentially evaluating its potential application in biological system.

**Figure 1:**
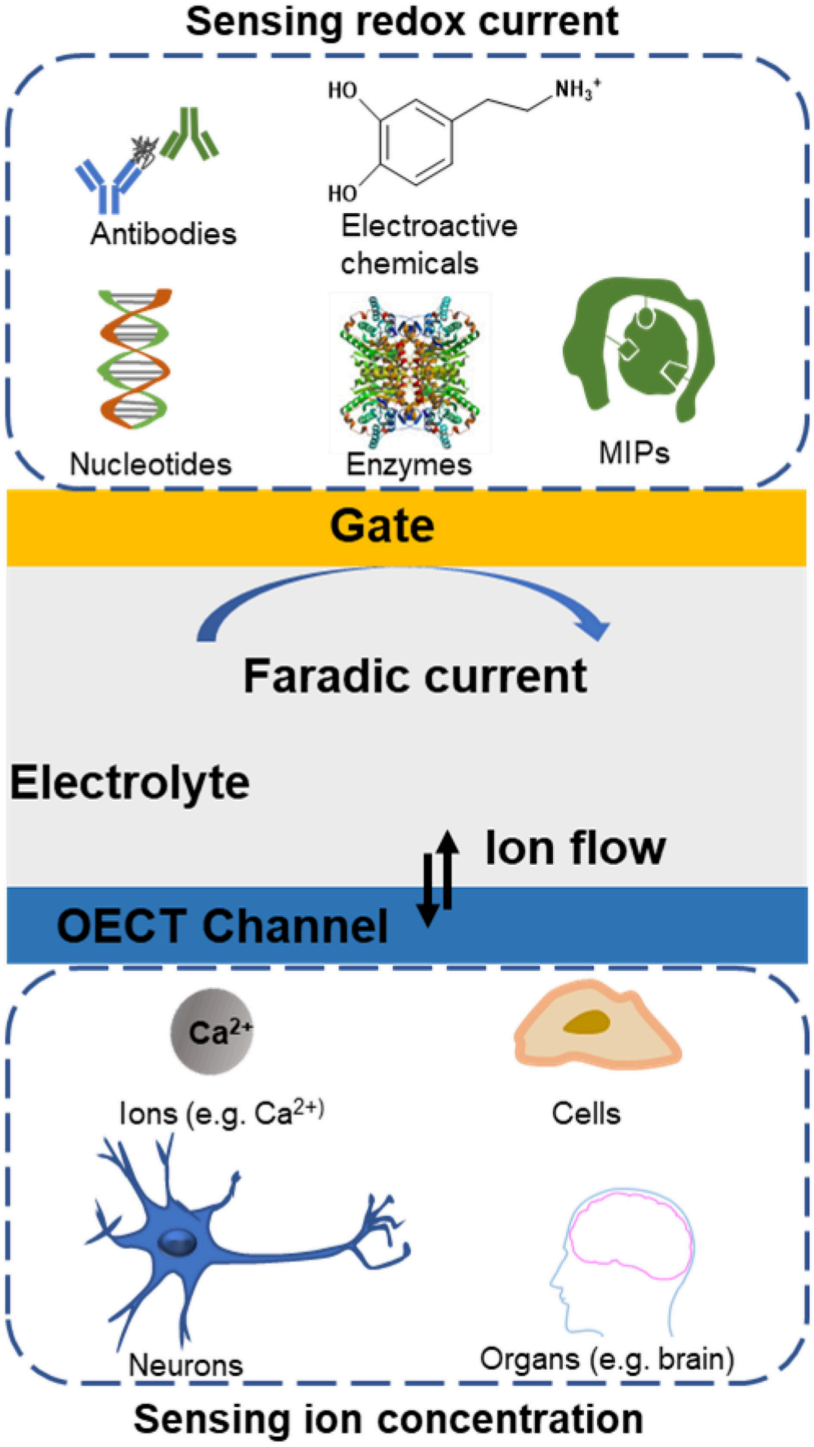
Overview of biological systems applications based on organic electrochemical transistors.

Based on these unique properties of OECTs, various applications have been explored during the last two decades (Figure 1), including neural interfaces (Khodagholy et al., 2013a, 2015, 2016; Campana et al., 2014; Yan et al., 2015), chemical and biological sensors (Nakatsuka et al., 2018), printed circuits (Zakhidov et al., 2011; Kim et al., 2013; Lee et al., 2017), neuromorphic devices (Uguz et al., 2016; Gkoupidenis et al., 2017), and clinical or biomedical researches (Berggren and Richter-Dahlfors, 2007; Rivnay et al., 2013; Someya et al., 2016). In electrophysiological signals, OECTs are used both as the recording and stimulation devices (Williamson et al., 2015; Braendlein et al., 2017a). OECTs can also be applied to measure cell coverage (Lin et al., 2010), barrier tissue arrangement and cellular environment for non-electrogenic epithelial cells (Jimison et al., 2012; Yao et al., 2013; Ramuz et al., 2015; Romeo et al., 2015). Assessment protocols have been established, applying white noise at the gate (Rivnay et al., 2015b), showing that OECTs have better performance comparing with impedance sensing (Rivnay et al., 2015b,c). The ability to work in complicated environments, such as blood and milk, paves the way for multi-analyte assay in complex environments (Tria et al., 2014). As for OECT-based biosensors, the detection of metabolites in electrolytes or body fluid are valuable for early prediction of human disease (Zhu et al., 2004; Macaya et al., 2007; Bernards et al., 2008). OECTs were easily to be coupled with various fabrication techniques (ElMahmoudy et al., 2017), resulting in different formative factors with flexible and wearable applications (Fan et al., 2018; Yang et al., 2018). The characteristics of ultrahigh transconductance (Khodagholy et al., 2013b), stability in electrolytes (Lee et al., 2018), cytocompatibility (Inal et al., 2016), and biofunctional modification (Someya et al., 2016; Curto et al., 2017; Pappa et al., 2017) result in their specially appropriate for bioelectronics fabrication. OECTs can also be prepared by conducting polymers compatible with cellular platforms, offering the possibility to modulate the bio-chemical, mechanical, and electrochemical microenvironment of cells and cell health where cells behavior can be concurrently monitored.

Some of previous reviews have summarized the physics and principles of OECTs (Rivnay et al., 2018), and the applications of OECTs on the organic bioelectronics (Simon et al., 2016; Someya et al., 2016), neural monitoring (Yan et al., 2015; Rivnay et al., 2017), and other biological applications (Xenofon et al., 2015). In this review, we mainly emphasize on the recent progress of OECT-based biological applications. Firstly, we would briefly introduce the configuration of OECT, working principle and general conducting polymer for OCET. Then, the biological applications, including biosensors and cell electrophysiological recording, would be discussed in detail. In each part, we will critically demonstrate the working mechanism and evaluate the unique properties for OECTs that facilitate the application progress for special requirements. Finally, challenges and opportunities still exist toward the biofunctional OECTs and the forthcoming studies are envisioned.

## Brief Introduction of OECTs

### Typical Configuration of OECTs

**Figure 2:**
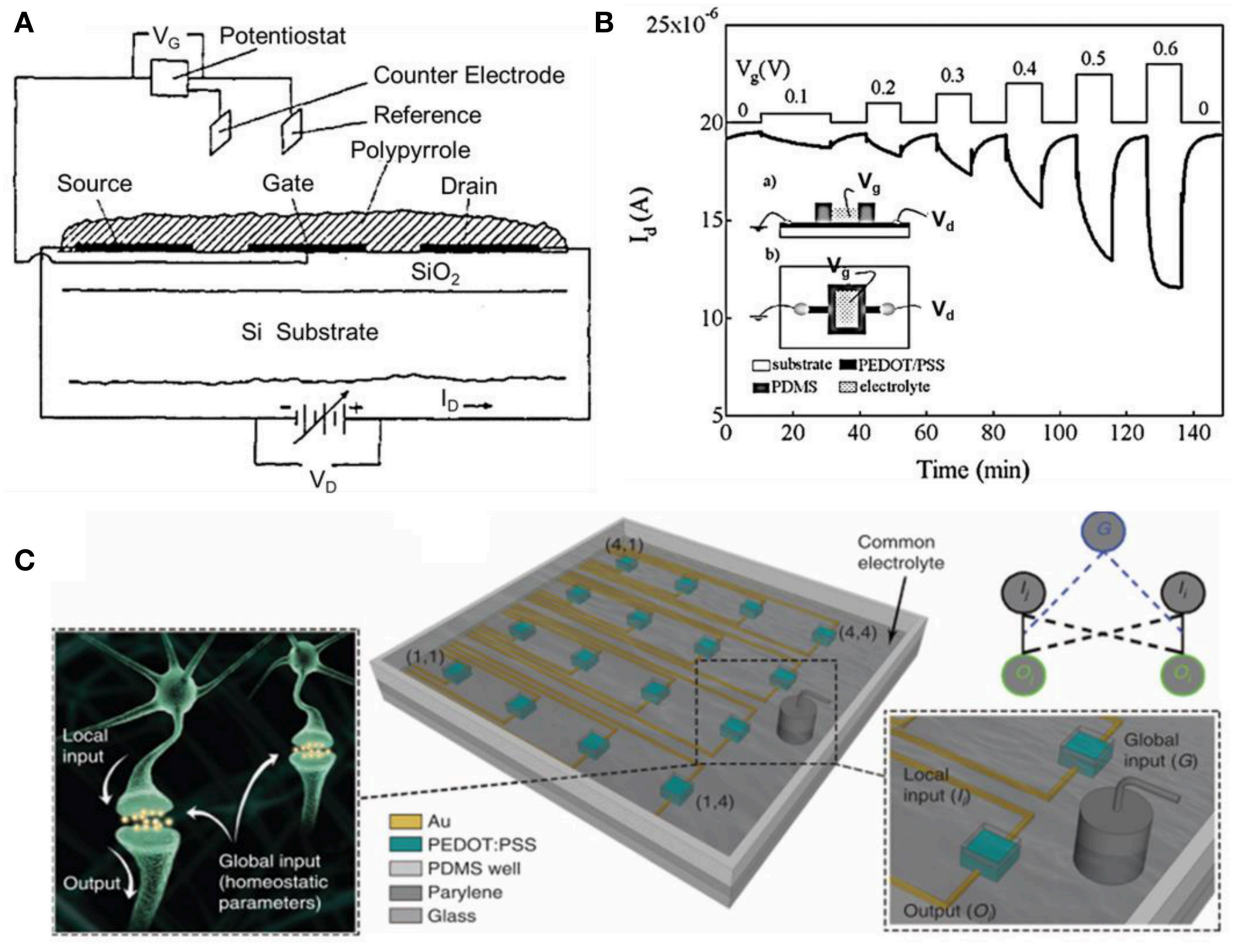
**(A)** Cross-section view of the array of three gold microelectrodes with polypyrrole fabricated transistor (White et al., 1984). Reproduced with permission (White et al., 1984). Copyright^©^ 1984 American Chemical Society. **(B)** Drain current-time response of PEDOT: PSS device. (Inset: the side view (a), and is top view (b) of the device) (Zhu et al., 2004). Reproduced with permission (Zhu et al., 2004). Copyright^©^ 2004 The Royal Society of Chemistry. **(C)** Illustration of PEDOT: PSS array, neurons are immersed in the same electrolyte and the analogy diagram of the device grid (Gkoupidenis et al., 2017). Reproduced with permission (Gkoupidenis et al., 2017). Copyright^©^ 2017 Nature Publishing Group.

The OECTs were firstly built by White et al. (1984), in which they reported a device with a microelectrode array that can work as a transistor to amplify the tiny current when it was immersed in an electrolyte solution (Figure 2A). In this work, they for the first time separated the gate electrode and the semiconductor channel with electrolyte, so that both the gate and the channel interfaces can be functionally modified. It is worth to note that the gate electrode was controlled by a traditional three-electrode electrochemical system in White's configuration as shown in Figure 2A (White et al., 1984; Nishizawa et al., 1992). In this configuration, the counter and reference electrodes are the essential components and they, respectively, establish the current circuits and ensure the stability of the potential especially for the cyclic potential scanning. If the gate potentials kept at constant value, the gate electrodes could be simplified into only one electrode because the voltage bias applied on the gate relative to the source electrode is constant, which would not influence the transfer or transconductance curve (Zhu et al., 2004). From then on, various devices based on the configuration of separated gate and channel with electrochemical and biological applications have sprung up. The most typical configuration of OECTs were constructed with one electrode (gate) immersing in the electrolyte and one channel connected by the semiconducting film with two metal electrodes (source and drain), the applied bias on the channel modulates holes or electrons moving in the semiconducting film (Figure 2B). In addition to the configuration of gate electrode, the development of the integration and printing technologies with OECTs enable large area computing and integration arrays of OECTs (van de Burgt et al., 2018) (Figure 2C).

### Principle of OECTs

**Figure 3:**
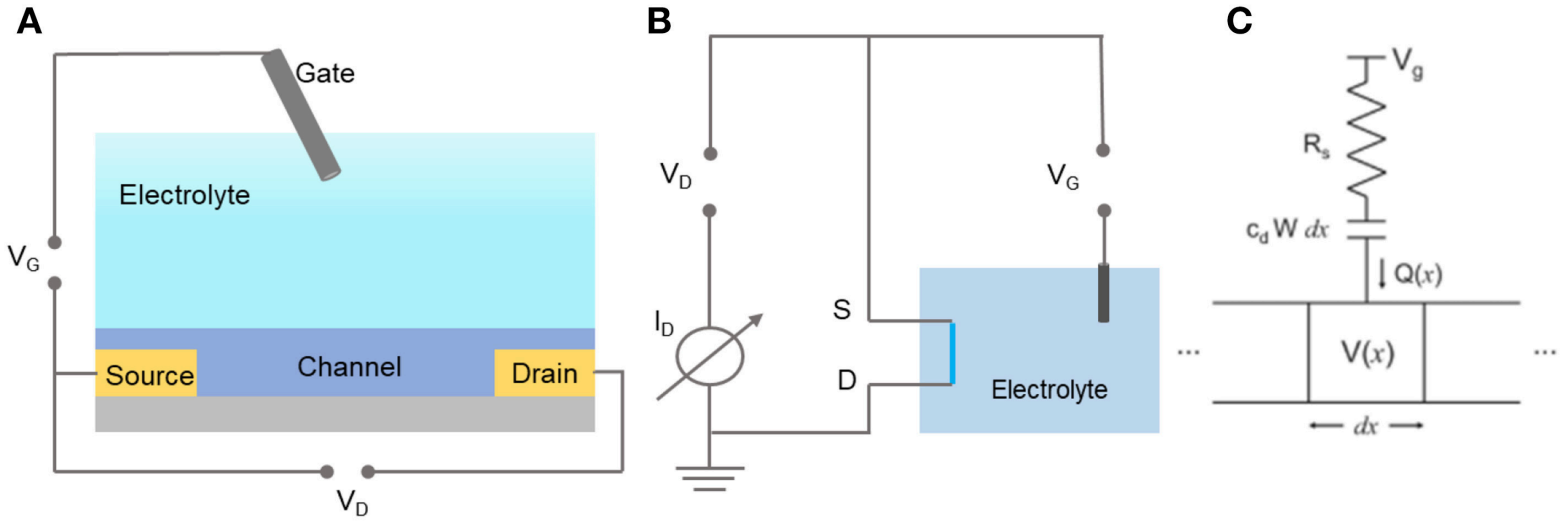
The schematic configuration of OECT sensor **(A)** and it's equivalent circuit **(B)**. **(C)** The typical ionic and elctronic circuit (Bernards and Malliaras, 2007). Reproduced with permission (Bernards and Malliaras, 2007). Copyright^©^ 2007 Wiley-VCH Verlag GmbH & Co. KGaA, Weinheim.

Bernards and Malliaras (2007) systematically demonstrated the steady-state and transient behavior of OECTs, which provided the initial view of the basic principle of OECTs. The principle was based on the ions in electrolyte transferred into the organic films, then its doping state and accordingly the conductivity of channel were changed. To operating the OECTs, the gate (gate voltage, V_G_) and the drain (drain voltage, V_D_) are controlled by applying constant voltages, which are referenced to the source electrode (Figures 3A,B). The potentials applied on gate electrode are related to the ions injecting into the channel and consequently controlling the doping state (i.e., redox state) of the conducting film. The drain current (I_D_), reveals the channel's doping state, which is proportionable to the quantity of mobile holes or electrons in the channel.

To qualitatively describe the working principle of OECT, The Bernards et al. postulates that there are two circuits of the OECTs: an ionic circuit and an electronic circuit, which separately describe the ions flow in the gate/electrolyte/channel system and the charges flow in the source/channel/drain system according to Ohm's law (Figure 3C). In the ionic circuit, ions flowing in the electrolyte is regarded as a resistor, and the ions volume in the channel is treated as a capacitor; while the electronic circuit is seen as a resistor. This model provides a prototype of the OECTs working principle, but there are still many significant properties that need to be taken into consideration. In this case, the influences of more factors to the efficiency of OECTs are conducted, such as, the thickness of the channel (Rivnay et al., 2015a), the mixed ion-electron transport (Rivnay et al., 2016; D'Angelo et al., 2018; Berggren et al., 2019; Onorato and Luscombe, 2019), and the double-layer (Tybrandt et al., 2017) coupling of conjugated polymers (Fan et al., 2019).

In addition, the Bernards' model demonstrates that both the ionic and the electronic circuit limit the response time. On the other hand, the efficiency of transduction, from small voltage signals on the gate to large current signals in the channel, is characterized with the first derivation of the transfer curve, called transconductance (g_m_ = ∂I_D_/∂V_G_). High transconductance values reflects the effective gating but leads to the slow operation of OECTs (Khodagholy et al., 2013b; Rivnay et al., 2013). In general, OECTs with the liquid electrolyte have the response time about tens of microseconds, which is suitable for most biosensor and for electrophysiological recordings (Rivnay et al., 2015c; Onorato and Luscombe, 2019). While the OECT with gel or solid electrolytes as ion transportation matrix is slower than that in liquid electrolytes (Bongo et al., 2013; Yi et al., 2015), which is more appropriate for the applications where a rapid response is not necessary.

With respect to the effective gating, the geometry of OECTs sensor plays an essential role (Cicoira et al., 2010; Lee and Someya, 2019). The geometry of the OECT includes the area ratio of channel and gate, the thickness of channel, and the distance between the gate and channel electrodes. OECTs with different area ratios of channel and gate will result in the following properties: (1) OECTs with small gates have smaller background noise; (2) devices show better sensitivity with small gates; (3) ΔI/I_0_ always becomes unchangeable at the same substrate concentration but not depend on channel/gate area; (4) the channel/gate area ratio is irrelevant to the detection linear (Hütter et al., 2013). The redox reactions produced by redox enzymatic active molecules, such as H_2_O_2_, control the potential drop at the interface of metal electrolyte/channel and gate/electrolyte (Bernards et al., 2008). Therefore, if the gate area for sensing application was small, the potential drop would be mainly on the interface of gate/electrolyte. And the reactions on the gate can be effectively amplified through the nature of transistor, and vice versa (Bernards et al., 2008).

### Conducting Polymers for OECTs

**Figure 4:**
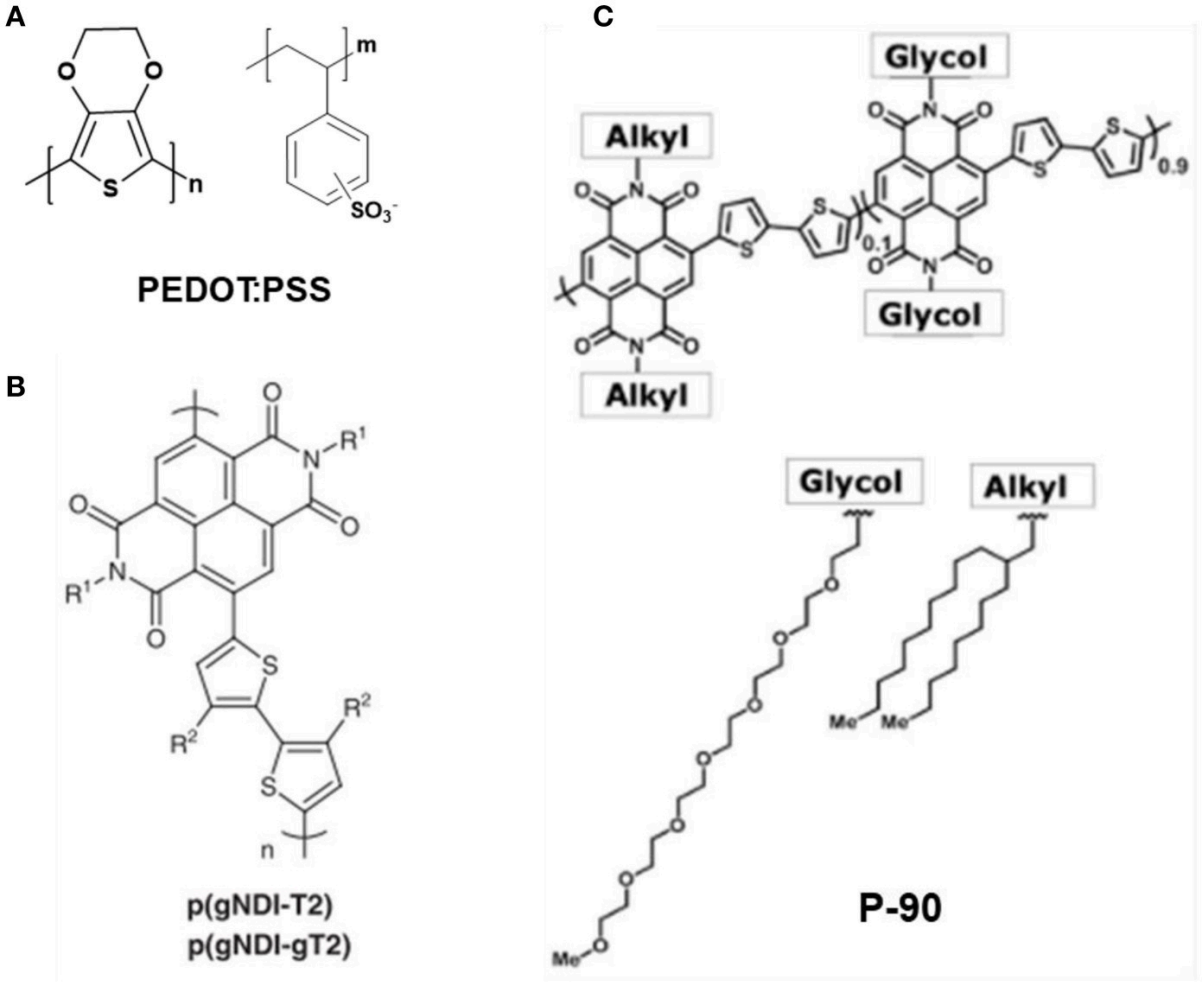
Chemical structure of typical conducting polymer: **(A)** PEDOT: PSS. **(B)** p(gNDI-T2) and p(gNDI-gT2) (Giovannitti et al., 2016). Reproduced with permission (Giovannitti et al., 2016). Copyright^©^ 2016 Nature Publishing Group. **(C)** P-90 (Pappa et al., 2018). Reproduced with permission (Pappa et al., 2018). Copyright^©^ 2018 American Association for the Advancement of Science.

The channel for OECTs is always constructed by organic semiconducting polymers which always bears excellent redox activity. So far, several kinds of conjugated conducting polymers of p-type (polythiophenes, polyfluorene, donor-receptor copolymer, etc.) and n-type (commonly based on copolymers of thiophene and fluorene) has been used for constructing OECTs. Among of them, the PEDOT: PSS (Figure 4A) is most common polymer as the semiconducting channel of OECTs. The PEDOT is p-type doped (oxidation state), therefore holes can hop among chains, so that applied positive bias on drain will produce the hole current. The sulfonate anions of PSS are added to stabilize the oxidized polymer PEDOT, to compensate for the shortage of negative charges. The p-type conducting polymers based OECTs always perform the depletion modes (ON state at the zero gate-source bias). While the n-type polymers working in accumulation modes are lagged behind the p-type, they are significant for preparing PN-junction and logic circuits, which is crucial for neuromorphic computing and bioelectronic applications. Most recently, n-type copolymer p(gNDI-T2) and p(gNDI-gT2) (Figure 4B) have been developed for OECT fabrication in aqueous electrolyte (Giovannitti et al., 2016), and P-90 (Figure 4C) is applied to detect lactate (Pappa et al., 2018). In addition, organic small molecule materials, such as pentacene, rubrene, fullerene, octa hydroxyquinoline, was also used as the semiconductor channel. However, the conjugated polymers always possess long strip and conjugated π or p-π structure, which will facilitate the preparation and readily built the thin film with low condition requirements. Therefore, the conjugated polymers are superior to organic small molecules not only for the excellent ability to conduct carriers (holes and electrons), but also their low-cost property and accessible fabrication (Flagg et al., 2019).

The particular identification of OECTs is the doping reactions appear among the whole volume of the organic film, which is different from FETs occur on a thin interfacial region. Therefore, low-gate voltages can achieve large modulations in the drain current, herein, OECTs are effective switches, and powerful amplifiers. In addition, compared with the solid dielectric layers, the electrolyte solutions are more applicable for larger flexible devices and various substrates' integration (Volkov et al., 2017; Fidanovski and Mawad, 2019). Moreover the intrinsic quality of the tunable organic molecules will further optimize the transport of ions and electrons and simplify the bio-interfaces (Giovannitti et al., 2016; Inal et al., 2016; Pappa et al., 2018; Sun et al., 2018). All these unique properties of OECTs essentially guaranty its wide applications in biological systems as demonstrated below.

## Biological Applications of OECTs

### Biological Sensing

In this section, we mainly introduced the operating mechanisms and reviewed recent applications of the OECT-based biological sensors. For electrochemical sensors, biomolecules in biological system could be classified into electroactive and electro-inactive species (Wu et al., 2017). The electroactive molecules (e.g., norepinephrine (NE), dopamine (DA), serotonin (5-HT), histamine, ascorbic acid (AA), uric acid (UA), CO, NO, H_2_S, etc.) can be directly oxidized or reduced on the electrode. While the electro-inactive molecules (e.g., glucose, glutamate, lactate, ATP, small molecule proteins, nucleic acid) can be detected by electrochemical technique through bio-recognition methods. Guided by changes of the potential drop on the gate and channel, the biosensors can generally be subdivided into the enzymatic sensing, immune-sensing, and aptamer sensing (Gentili et al., 2018). Other kinds of OECT-based sensors utilizing the changes of doping state of the conducting polymer altered by the factors, such as ions concentration and pH, are essential to advocate the progress of OECTs biological sensing (Qing et al., 2019).

**Figure 5:**
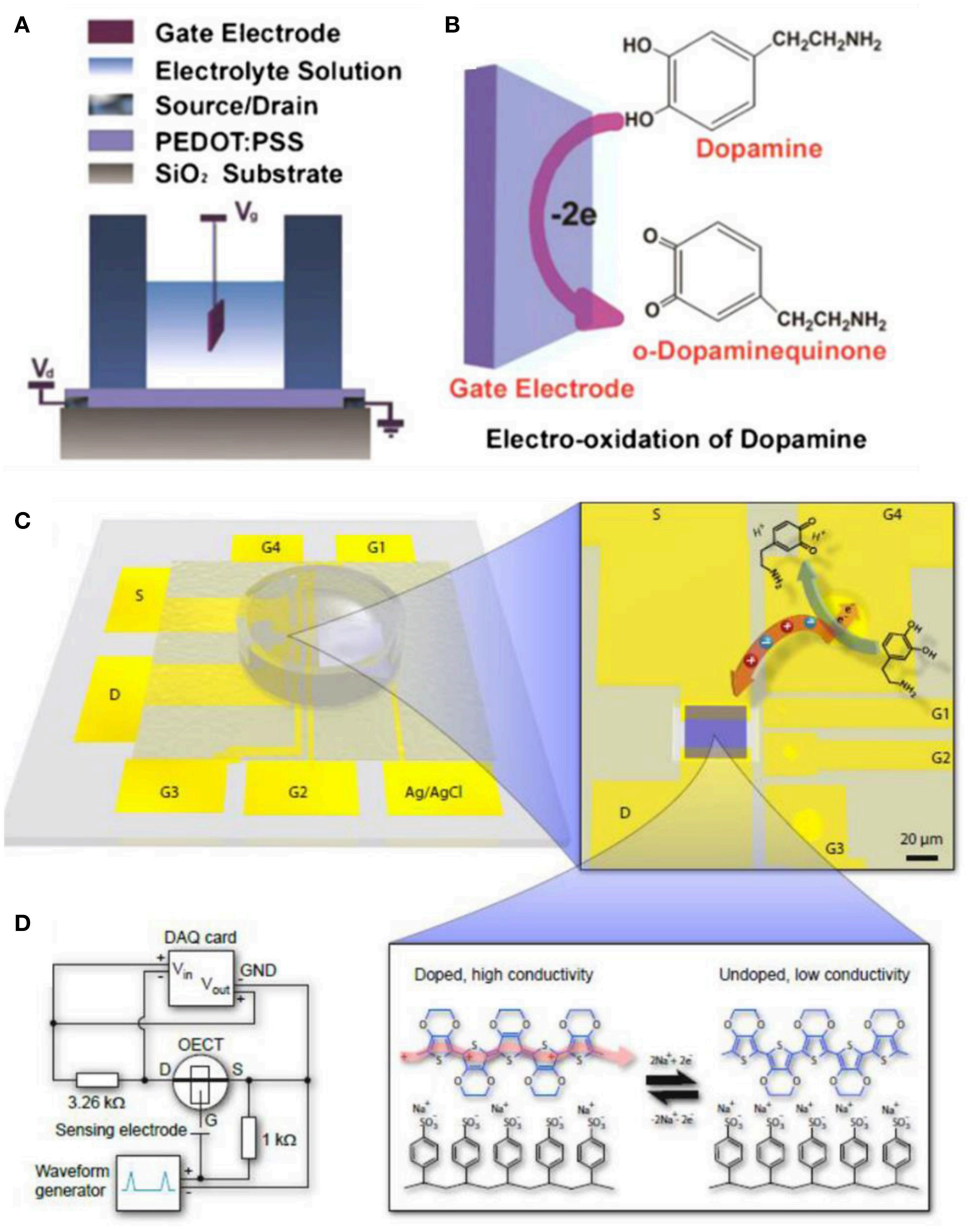
**(A)** Illustration of OECT-based dopamine biosensor that PEDOT: PSS act as the conducting channel. **(B)** The oxidization of dopamine on the surface of the gate electrode (Tang et al., 2011). **(A,B)** reproduced with permission (Tang et al., 2011). Copyright^©^ 2011 Elsevier B.V. All rights reserved. **(C)** The OECT device and gate electrodes built by patterned gold. And the illustration of dopamine oxidized on the Au gate shifting the doping state of the PEDOT: PSS channel **(D)** Experimental device circuit used for the OECT-amplified FSCV (Tybrandt et al., 2014). **(C,D)** reproduced with permission (Tybrandt et al., 2014). Copyright^©^ 2014 Elsevier.

The electroactive chemicals (e.g., DA, E, NE, 5-HT) in biological system, can be directly oxidized or reduced on the electrodes. For example, DA can be oxidized to o-Dopamine quinone (Figures 5A,B) at a certain voltage. The OECTs as a kind of transistors possesses the characteristics of amplifying in-put signals, while coupling with the nanomaterials technology, the OECT-based sensors will result in significant sensitivity improvement (Liao et al., 2014; Mak et al., 2015). In this case, the construction of the high-performance gate electrode is crucial for high-sensitive sensing. Tang et al. (2011) built a dopamine OECT sensor by comparing five kinds of gate electrodes (including graphite, Au and Pt electrode, etc), and found that the Pt gate bears the highest sensitivity as to detect 5 nM DA at the potential of 0.6 V. The synergistic effect of gate and channel for redox signals transduction will further increase the sensitivity. Gualandi et al. built an all-PEDOT: PSS OECT to sensing AA (Gualandi et al., 2015), in which by modulating the V_G_ and film thickness, the detection limit can be reach to 10^−8^ M level.

Selectivity is another important parameter for OECT-based sensors, especially for complicated samples. Gualandi et al. (2016) constructed an all-PEDOT: PSS-based OECT, which got the separated transconductance (g_m_) linear calibration of DA without the interference from AA and UA, through changing the scan rates and gate voltages, in which case, the scan rate is an essential property for sensitivity. Classic electrochemical methods cooperate with OECT will promote the development of biosensing: fast scan cyclic voltammetry (FSCV) is widely used to detect electroactive chemicals, especially DA in neuro system, (Tybrandt et al., 2014) combined the FSCV technology and OECT (Figures 5C,D) successfully amplified the small detected signals under no shielding environment. This integration paves the way for multifunctional bioelectronics applying in various devices (Sivakumarasamy et al., 2018).

#### OECTs Based Enzymatic Biosensors

The most widely used OECTs biosensors are coupled with enzymes. The first application of the OECTs interfacing with enzymes was based on the device model developed by Nishizawa et al. (1992). They detected the conductivity of the polypyrrole channel on account of the film's responsiveness to pH induced by the penicillinase enzyme reaction. However, the polypyrrole film is pH-dependent and the linearity range is limited by the thickness of polymers, losing the regulating ability in neural pH environments. Zhu et al. (2004) illustrated a PEDOT: PSS-based transistor for glucose sensing in neutral pH electrolytes. Since PEDOT: PSS is stable in a wide pH range, it is applicable in the enzymatic sensing under neutral environment. Moreover, the bias potentials between gate and channel are small so there exists no hydrolysis of electrolyte, and the known initial properties of the transistor are enough to meet the need for detecting several analytes, from then on, PEDOT: PSS plays an important role in the OECTs enzymic sensors.

**Figure 6:**
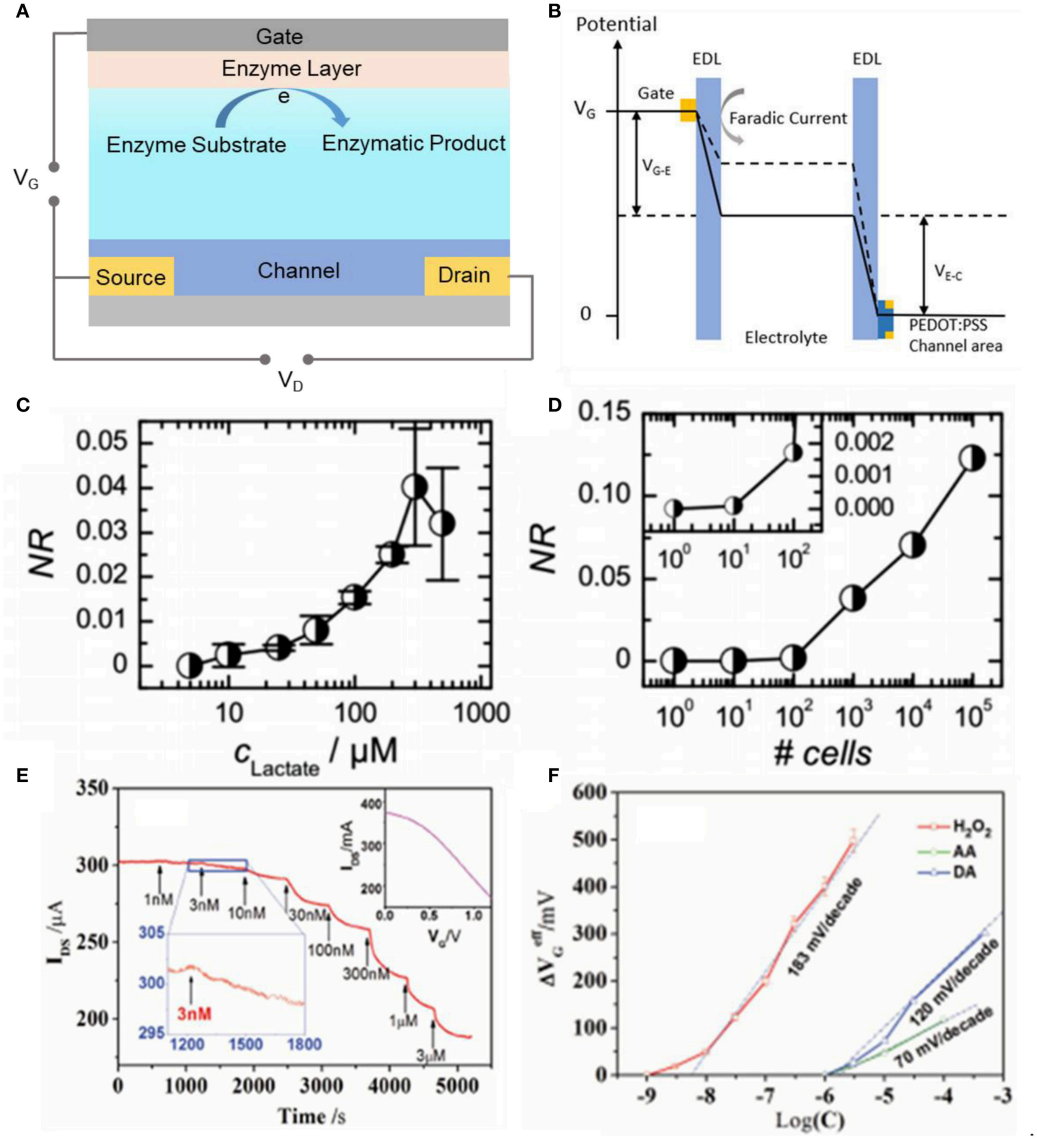
Schematic working principle of an enzyme modified OECT sensor of **(A)** the reaction on the enzymatic gate electrode and **(B)** typical potential drop between the channel and gate with the addition of substrate. Characteristics of the sensitivity **(C)** of lactate sensor of three devices [NR (normalized response) = ΔV _out_/ΔV _out, max_] and **(D)** titration curve of one selected device with consecutive addition of substrate collected from cells culture of different concentrations (Braendlein et al., 2017b). Reproduced with permission (Braendlein et al., 2017b). Copyright^©^ 2017 WILEY-VCH Verlag GmbH & Co. KGaA, Weinheim. **(E)** Titration curve of an OECT modified with PANI/Nafion-graphene/Pt as gate electrode to the additions of H_2_O_2_. Inset: transfer curve of the device (Liao et al., 2015). **(F)** The effective gate voltage (ΔV _G_^eff^) vs. different concentrations of H_2_O_2_, AA, and DA (Liao et al., 2015). Reproduced with permission (Liao et al., 2015). Copyright^©^ 2014 WILEY-VCH Verlag GmbH & Co. KGaA, Weinheim.

The sensing mechanism of the typical enzymatic OECTs sensors is shown in (Figures 6A,B): Enzymes are immobilized on the gate electrode and catalyze the substrates into enzymatic product, through which they obtain or lose electrons on the electrode, the electrical signals are transferred to the gate electrode and further leading to channel current changes simultaneously. To maintain the charge neutrality in the whole circuit, including the ionic circuit and electronic circuit, a cation penetrates into the conducting polymer film and replaces the role of the cationic polymer (e.g., PEDOT^+^) compensating the anionic polymer (e.g., PSS^−^), which leads to a change of the V_g_^eff^, then a decrease of the channel current which is logarithmically proportional to concentration of enzymatic substrates.

The sensitivity of OECTs devices, lies on the nature of amplification provided by the optimized geometry and the sensitive enzymatic modification. After optimization using new materials and different modification technology, the detection levels of bioactive molecules can be promoted to a large extent. Apart from the mostly used p-type conjugated polymer, Pappa et al. (2016) demonstrated a new kind of OECTs enzymatic sensor by using an n-type conducting polymer in accumulation mode. The n-type conjugated polymer is based on the NDI-T2 polymer (also called P-90). The side glycol chains ensure the solubility of the polymer in an electrolyte and enable the enzymes anchored on the polymer and strengthen polymer's water absorbability. By modifying LOx on both channel and gate, the detection concentration of lactate can low to 10 μM, while still can't overmatch with nM level of PEDOT: PSS modified OECTs.

In addition to the high sensitivity, selectivity is another key role for the biological sensors. So far, there are several methods to improve the selectivity by rationally designing the surface chemistry of gate electrode (e.g., modifying special materials and/or modulating the interaction of ions (Yu et al., 2015). Liao et al.(Figures 6E,F) (Liao et al., 2015), reported a highly selective OECTs-based enzyme sensors. By modifying a bilayer film on the gate electrode, the interference of charged molecules coexisting in the solution with the analytes effectively decreased. The high selectivity of the device in this work is accomplished by firstly covering a thin layer of the compound graphene flakes and Nafion, then a thin layer of polyaniline (PANI) polymer on the gate electrode. The graphene flakes in the film are used to enhance the electrochemical catalytic performance and the conductance of gate electrode, the PANI film carrying positive charge can repel the positively charged bioactive molecules as NE, and Nafion film that is negatively charged can hinder the anionic electroactive molecules like AA and UA.

The outstanding performance of the biosensors compromise the reaction both on the gate and the channel, thus they have equivalent ability to tune the potential drop on the electrode-electrolyte interfaces, receiving the amplified signal. Wang's group (Wang et al., 2017), successfully built an OECTs glucose biosensor based on a novel woven fiber composed of polypyrrole (PPy) nanowires and reduced graphene oxide (rGO). They reported that the addition of rGO nanosheets will enhance the electronic performance of the fiber electrodes. The composites-based transistors exhibit high switch capability, fast switch speed, and long durability under electrical experiments. The transistor exhibits remarkably sensitivity for glucose sensing, the NCR/decade can be reach to 0.773. Besides, the sensors show the fast response time (i.e.g,0.5 s), good reversibility, wide linear range of glucose concentration and a lower detection limit compared with conventional methods.

Moreover, the OECTs can be used for multianalyte detections by combination with the array techniques. Pappa et al. (2016), developed a multianalyte biosensing platform using the OECTs array. The device is able to sense three typical clinically related molecules, such as glucose, lactate, and cholesterol by immobilizing relative glucose oxidase (GOx), lactate oxidase (LOx), and cholesterol oxidase (ChOx) on the PEDOT: PSS coated gate electrodes, without the electrical and chemical cross-talk over different transistors. This biosensor could be used for real sample analysis in saliva. Braendlein et al. (2017b), introduced a reference-based sensor circuit, through compromising two OECTs functionally differently, used the popular p-type semiconductor PEDOT: PSS, toward a Wheatstone bridge design. One of the two OECTs is used as the reference (immobilized with BSA) and the other is functionalized (immobilized with lactate oxidase) as the indicator. The sensor shows a low limit of detection generated by cells, estimated to be ≈10 × 10^−6^ M, and the reference lactate concentration of fresh cells is assessed to be 50 × 10^−6^ M (Figures 6C,D). Such a circuit was firstly applied to clinically relevant testing and significantly help to accurately diagnose preliminary tumor treatment.

Disposability and stability are another key performance for the biosensors, which are benefit from the application of multifunctional materials and simple configuration techniques. Shim et al. (2009), demonstrated an all-PEDOT: PSS OECT-based glucose biosensor by introducing the mediator (e.g., ferrocene) to transfer generated electrons of redox reactions to the gate. This facile fabrication of OECT-based enzymatic biosensors realizes the character of low-cost. Khodagholy et al. (2012), incorporating ionic liquids and conjugated polymers, constructed an OECTs-based lactate biosensor by solid state gel, which contains: lactate oxidase and the mediator ferrocene for shuttling the electron transfer. This type of device is a promising wearable sensor for continuously monitoring lactate levels in athletes. Bihar et al. (2016), built an OECT-breathalyzer, with the conducting polymer PEDOT: PSS printed on paper, and the enzymatic reaction occurred in the electrolyte gel. This device is easy to use for the low-cost and disposable portable sensors to facilitate blood alcohol content sensing.

#### OECTs Immunosensors

Immunoassay is crucial in clinical analysis (cancer cells, biomarkers, pharmaceuticals), toxins, and microbials in the environments (Wen et al., 2017). Antibodies of the immune system are regarded as the biorecognition element, and lead to the high specificity and sensitivity of the immunosensors. As to the immune sensing, the competitive and sandwich types are widely accepted. For competition reactions, the immobilized biorecognition molecule can be either an antibody or an antigen. The sandwich-type immunosensor consists of a fundamental antibody fixed on the platform of a sensor, and the specific antigen marker in a sample solution, the secondary antibody reacts with the antigen bound, then the detectable and low noise signal are generated via enzymes (Zhang and Heller, 2005; Yu et al., 2006), redox substrates (Viswanathan et al., 2009), or nanomaterials (Liu and Lin, 2007; Kerman et al., 2008). Based on this principle, Kim et al. (2010) demonstrated an immunosensor based on OECT to sense prostate specific antigen (PSA), through conjugating AuNPs with PSA specific antibody on the conducting channel. The limit of detection was low to 1 pg/mL compared with the maximum detection value of 4 ng/mL. Torsi's group has developed various OECT-based immunosensors transistors. In their recent study, they developed the anti-human Immunoglobulin G (anti-IgG) sensor at the detecting limit of femto-molar (Macchia et al., 2017). The anti-IgG OECT sensors have the ability to detect IgG with high biomolecular interaction in the femtomolar (fM) range by immobilizing the anti-IgG on the gold gate. They also showed a plastic OECT sensor (Macchia et al., 2018) by the gate modification, which possess the low-cost, ultra-sensitive properties, and further paves the way for application of immunoassay technology. Fu et al. (2017) have successfully applied OECTs to the sensing of protein by detecting protein cancer biomarkers in cells. The gate is modified with antibodies and catalytic nanoprobes so that the gate electrode can capture the target and output the current response for the product of catalytic reaction (i.e., electroactive H_2_O_2_). For the amplification nature of organic electrochemical transistors, the biosensor that detect the cancer marker HER2 has the detection limit low to 10^−14^g mL^−1^, which is several magnitudes lower than original electrochemical methods. Moreover, the OECTs based HER2 biosensors have a detecting concentration range from 10^−14^ to 10^−7^g ml^−1^, which covers the detect amount of HER2 in normal and breast cancer cells. Additionally, these protein sensors can distinguish the cancer cells from the breast cancer cells attribute to the specificity of the modified antibody.

#### OECTs Aptamer Sensors

Aptamer-based technology bearing the high specificity or even superior to antibodies, that has the potential for applications of diagnostics and therapy. DNA sensor based on OECT has been fabricated by Lin et al. (2011), they integrated the OECT with a flexible microfluidic device. The label-free DNA sensor was fabricated with single stranded DNA which was immobilized on the gate as the DNA probe to detect the complementary DNA targets, and the detection limit was 1 nM. More importantly, the detection limit could be extended to 10 pM if an electric pulse was applied to the gate to increase the hybridization of DNA. The modulation mechanism is the changes of surface charge on the gate electrode generated by hybridizing DNA. They hypothesized that the thickness of the electrolyte double layer is much thicker than the DNA layer, therefore, the potential of gate is not affected by the concentration of ions in the electrolyte. Peng et al. (2018) have built an OECT sensor for detecting microRNA21 by using the Au NPs and capturing probe protein to modify gate electrode. The amplification effect of OECTs and the immobilized Au NPs, leading to the high sensitivity, selectivity, and acceptable applicability of microRNA21 assay in total RNA sample, which provides potential application in the future microRNA analysis.

#### OECTs Coupled With Artificial Receptors

**Figure 7:**
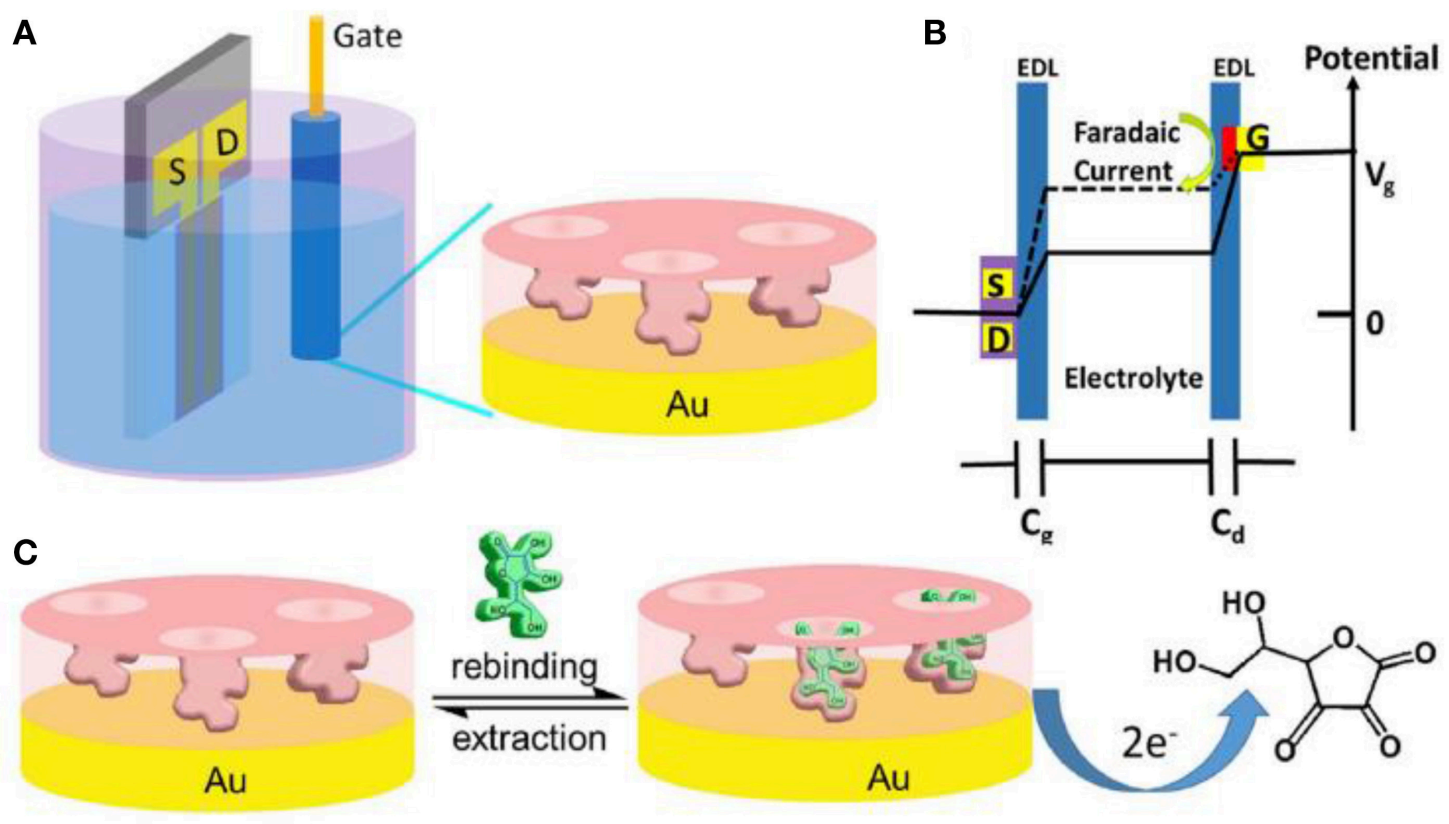
**(A)** Schematic diagram of an AA sensor based on the MIP modified OECT. **(B)** Potential drop between the channel and gate of the OECT. **(C)** Schematic for the sensing of AA by MIP film modified gate (Zhang et al., 2018). Reproduced with permission (Zhang et al., 2018). Copyright^©^ 2017 Elsevier B.V. All rights reserved.

Artificial receptors possess comparatively higher chemical stability than natural receptors under physical environments, consequently they provide possible alternatives on widespread biological applications (Labib et al., 2016). Very recently, molecularly imprinted polymers (MIPs) shows great potential for the development of biotechnology, diagnostics, and stretchable devices. Parlak et al. developed an artificial receptor based wearable OECT device by preparing the molecularly selective membrane for cortisol detection (Parlak et al., 2018). The artificial receptors can overcome the instability of natural biomolecules, such as enzymes and antibodies. The biomimetic membrane based OECT was configured with wearable substrates and sample reservoirs, this device can readily get the stable signals reading-out by collecting enough sweat about 10 to 50 μL. The MIP were entrapped into a plasticized poly (vinyl chloride) (PVC), which acts as an ionic barrier to decrease the g_m_ but still maintain the efficient OECT capability. And the device was applied *in vitro* and on body to conduct the cortisol changes by measuring electrochemical performance. It's meaningful that the device was used in exercising human to detect the cortisol concentration. Zhang et al. (2018) combined the molecularly imprinted polymers (MIP) with OECT-based sensor to detect ascorbic acid (AA) (Figure 7). The selectivity of the MIP films on the gate was conducted by the preparation of the polymer with AA removal and rebinding on the surface, then the polymer film acted as the recognition unit of the sensor. They found various species, such as H_2_O_2_, Gly, GSH, UA, Glu, Na^+^, K^+^, Fe^2+^, Mg^2+^, Ca^2+^, and ASP, that has almost no interferences for AA detection. The sensors could be used for the AA analysis of vitamin C beverages.

#### OECTs Ion Sensing

The OECTs can be used for ion detection due to the ions transfer freely in the whole bulk of electrolyte, which includes not only aqueous solution but also other ionic and electronic media, such as gel and ionic liquid (Duc et al., 2017; Dai et al., 2018). Recently, Gentili et al. (2018) demonstrated a new principle of current-driven inverter-like, low-voltage, high-sensitivity ion detection OECT. Differently with the voltage-driven OECTs, the current-driven OECT configuration provides, low-voltage operation and high sensitivity, where the sensitivity depends on the large g_m_ and load resistor. However, the applied voltage should consistent with the physiological environment. So that the trade-off among sensitivity, operating range, and applied voltage are needed. The current-driven OECT configuration is based on the recording of the changing voltage generated by the ions concentration, differentiating it from the usual ion sensors where the changing of ion concentration transferred as the output voltage. Consequently, the current-driven OECT study take advantage of the large g_m_ of OECTs in an absolutely different way in relative to regular voltage amplifier structures, which introduce a new kind of trade-off between sensitivity and working voltage. While (Del Agua et al., 2018) introduced a PEG-based Na^+^ conducting hydrogel as OECT electrolyte. The hydrogel is prepared by fast photopolymerization with commercial monomer. And the hydrogel has high ionic conductivity and can be contained during the fabrication of photolithography device. With the high performances at room temperature, this hydrogel has the possibility to replace liquid electrolytes in versatile OECTs and accomplish print integrated into flexible OECT devices.

#### OECTs Cell Monitoring

**Figure 8:**
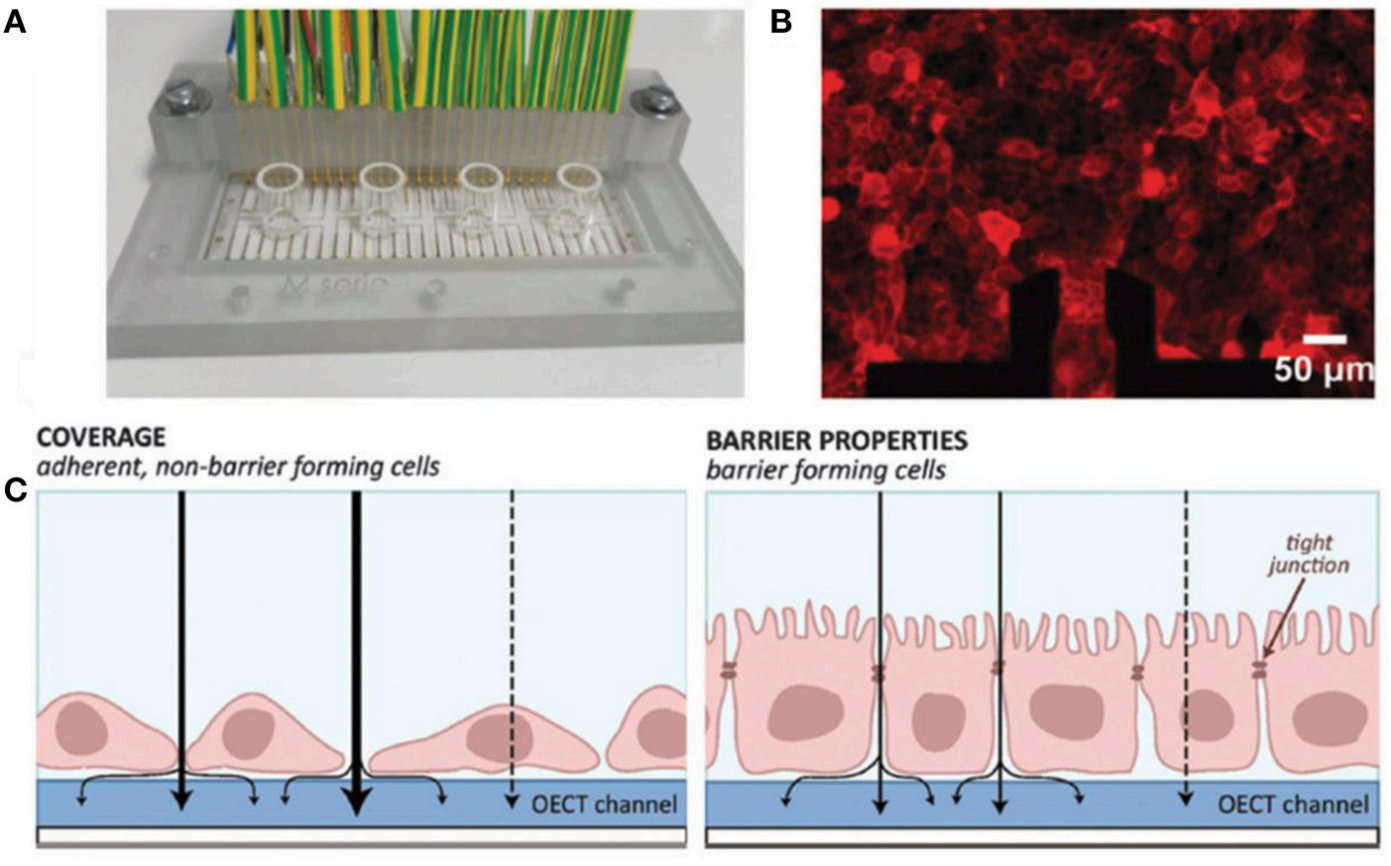
The OECT device for monitoring adherent cells. **(A)** Measurement platform consisting of 24 OECTs divided between four glass wells. **(B)** MDCK II cells transfected with RFP actin construct seeded on device for fluorescence imaging. **(C)** Schematic diagram of the cell coverage with low ion flow through barrier (right) or high ion flow through non-barrier (left) (Ramuz et al., 2015). Reproduced with permission (Ramuz et al., 2015). Copyright^©^ 2015 The Royal Society of Chemistry.

The coupling of OECTs with live mammalian cells for monitoring toxicology/diagnostics and other properties was developed in the past decade. Bolin et al. (2009) firstly coupled the OECTs with cells and detected the gradients of cells on the OECT channel. They seeded Madin Darby canine kidney (MDCK) epithelial cells on an OECT channel and the channel bias added and produced a potential gradient. The gate potential controls and modulates the potential gradient of channel. Therefore, the MDCK cell quantity gradient on the channel depended on the gate and source voltages. Lin et al. (2010) reported sensors based on OECTs combined with cancer cells and fibroblasts for sensing *in-vitro* cell activities. The sensing principle is the electrostatic actions on the interfaces of the cells and the OECT reactive layer. Since the device is sensitive to the surface charge changes induced by the adhesion of cells, it's applicable for solution processing to miniature and integrate cell-based sensors can further promote the cell relevant testing, such as screening drugs and testing toxic substances. The potential can predict the adhesion of cell and formation of epithelium (Gu et al., 2019). Ramuz et al. (2015) found that barrier tissue cells adhered on the polymer surface can be deprived of function in calcium switch assay, and re-addition of calcium lead to improvement of the cells function. The process is monitored both electronically and optically, enabling the capture of cells images while simultaneously recording electronic information (Figure 8).

Wei et al. (2017) presented the first report to apply OECTs for detecting the microalgae H. pluvialis cell. The constructed OECT array is a platform with advantages of convenience, and efficiency, and is able to monitor the real-time signals induced by settling H. pluvialis cells on the active conducting polymers. The results can help to estimate the time point approximately for producing the maximum astaxanthin in the commercial fermentation. Rivnay et al. (2015c), combined OECT with the impedance sensing technique by applying gate current to generate complicate low error impedance signals. They applied the method *in vitro* to sense a layer of epithelial tissue and concluded that the data is suitable to an equivalent circuit, allowing the resistance of trans-epithelial, and capacitance values of cell layer in accordance with literatures. Very recently, Chen et al. (2018) reported sensitive glycan sensors based on OECTs to detect the glycan on cells surface. PEDOT: PSS as the channel and the concanavalin A (Con A) loaded by poly dimethyl diallyl ammonium chloride (PDDA)–multiwall carbon nanotube (MWCNTs) modified the gate, which can correctly connect mannose by capturing the cancer cell. Specialized mannose nanoprobes are equipped by binding horseradish peroxidase (HRP) and aptamers on gold nanoparticles, then HRP-aptamer-Au NPs can be used to identify the human-breast cancer (MCF-7) cells, the prepared OECT-based glycan sensor leverage the electrochemical reaction on the gate electrode with the mannose on surface of captured cells. The HRP-catalyzed reduction to MCF-7 cells on the gate induced channel current responses, which generated H_2_O_2_ even when the cell concentration is low to 10 cells μL^−1^. To prove the specific recognition reaction, they added N-glycan inhibitor stimulation and the signal is dramatically decreased for the decrease of mannose on cells. Furthermore, the as-formed device can be applied to analyze other glycans and cancer cells by simply altering the binding lectins and aptamer sequences. This strategy paves the way for various glycans analysis on a cell surface.

### Neural Recording and Stimulation With OECTs

Recording electric signals of brain has been the most important but tough challenge in the past few decades since many kinds of diseases are strongly related to the change of signals between neurons. With the flourishing of semiconducting polymers and electrochemical transistors in the recent years, neural recording and stimulation get more opportunities to detect more precisely and applicable for more complicated *in vivo* sensing. This section focuses on the recent progress on the neural recording and stimulation based on OECTs, mainly including the development history, working mechanisms, and recent applications, and illustrate the enhanced performance for the multifunctional transistors.

#### Brief Introduction

In the brain, information transfer is achieved by the adjoining neurons generating bioelectrical signals, named action potential, propagating over the synapses. In the last decade, electric recording and stimulation based on metal electrodes have extremely conductive to our fundamental cognizing of real neural activities (Gilletti and Muthuswamy, 2006; Rivnay et al., 2017; Xiao et al., 2017). Conventional small metal or carbon-based microelectrodes are able to probe and stimulate neural activities with high resolution at the level of single-cell (Jasper et al., 1961). The metal microelectrode arrays can record the activities of a large population of neurons at the same time (Lee et al., 2017). Nowadays, implanting stimulating electrodes into the brain is progressively applied to deal with neurological disorders (Leleux et al., 2014; Lee et al., 2018). To compensate for available methods, the bioelectronics can be implanted into deep brain and conduct high temporal resolution, capable of directly communicating with the neuro net through electronics, and transduce neuron's electronic signals from bioelectronic signals (Jonsson et al., 2015). However, transformation presently challenged by the matching ability and electrons transfer on neural interfaces. For that metals transmit electrons signals while neural systems transmit ionic signals, the signal-to-noise signals (SNR) recorded and stimulated by bioelectronics are almost depended on the coupling transmission of electrons and ions on the metal/neural interfaces. Generally used metal electrode arrays are made of hard materials, such as gold, with their elastic module distinctly exceed the neural tissue in the range of kPa to MPa (Gilletti and Muthuswamy, 2006). The implanted electrodes in the neural tissues will cause inflammatory response results from the mechanical incompatibility, which will eventually result in electrode failure in long-term studies. In order to obtain favorable neuron/electrode interfaces, novel materials, organic conducting polymers-based bioelectronics, has exhibit strengthen neural recording and stimulation characteristics along with their merits of biocompatibility and low mechanical strength.

#### Mechanisms

**Figure 9:**
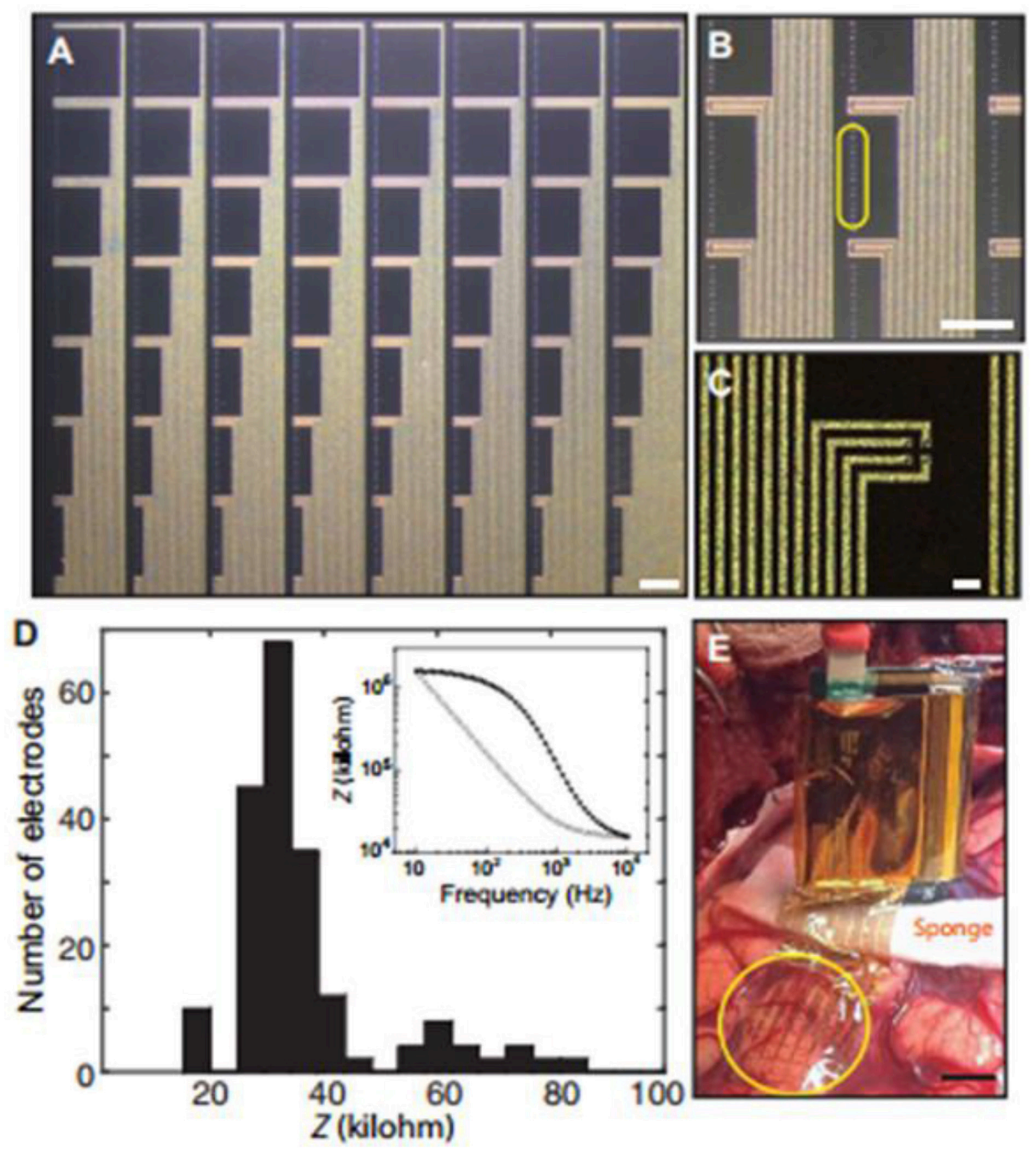
Device structure and characterization of NeuroGrid for intraoperative recording. **(A)** Photo of NeuroGrid (240 channel, scale bar, 1 mm). **(B)** Amplified picture of electrodes arranged in 2 × 2 tetrodes (2 mm spacing between each tetrode, scale bar, 1 mm). **(C)** Structure of electrodes coated by conducting polymer involving a single tetrode (scale bar, 20 mm). **(D)** Histogram displaying impedance of a single NeuroGrid: yield = 87.5% (240 electrodes involved). Inset: electrochemical impedance phase and magnitude of a single electrode at the level of physiological frequencies. **(E)** Photograph of intraoperation by putting NeuroGrid (240-channel, yellow circle, scale bar, 1 cm) on the surface of the cortex (Khodagholy et al., 2016). Copyright^©^ 2016 American Association for the Advancement of Science.

The mechanisms of neural recording by metal electrodes or transistors is explained hereunder: the active potential of a neuron, contributes to ionic currents flux over the cell membrane, which changes the cell membrane potential, then the potential results in an electrochemical signal on the interface of metal/electrolyte or changes potential-drop of the conducting polymer. While in the brain, an enormous neural network system, the total neural electric currents generated from numerous neurons in a small bulk at a specified area will generate local field potential (LFP). This LFP called the electrocorticography (ECoG) when using the electrode on the cortical surface, and when recording with the electrode inserted deep into the brain called stereoelectroencephalography (SEEG). The longer distance between the recording electrode and the location leads to useless signals for understanding the processes of neuropathology, hence the SEEG conduct the most informative signals. Conducting polymer-based electrode has extensively enhanced the electrical performances and biocompatibility of mental inserted intracortical electrodes. However, compared with the SEEG, the ECoG is non-invasive, the characteristics of ECoG have been significantly improved by using more stable polymers and rational building of devices (Khodagholy et al., 2016) (Figure 9).

As for the neural stimulation, charges flow from electrodes to neurons, and the charge injection ability on the electrode/neural interfaces is regarded as the parameters to be measured (Lee and Someya, 2019). The neural stimulation generated charges have the quantity that many orders of magnitude higher than the electrodes recorded signals, which mostly because of the meaningless coupling on the electrode/neuron interface. The ideal stimulation electrodes would be small enough to selectively put on the target. However, the small electrode requires high potential which leads to undesirable reactions and confuses the recording signals. Therefore, organic conducting polymers advanced the neural recording and stimulation, promoted OECTs application in the field of neural recording (Green et al., 2013).

#### Recent Advances

**Figure 10:**
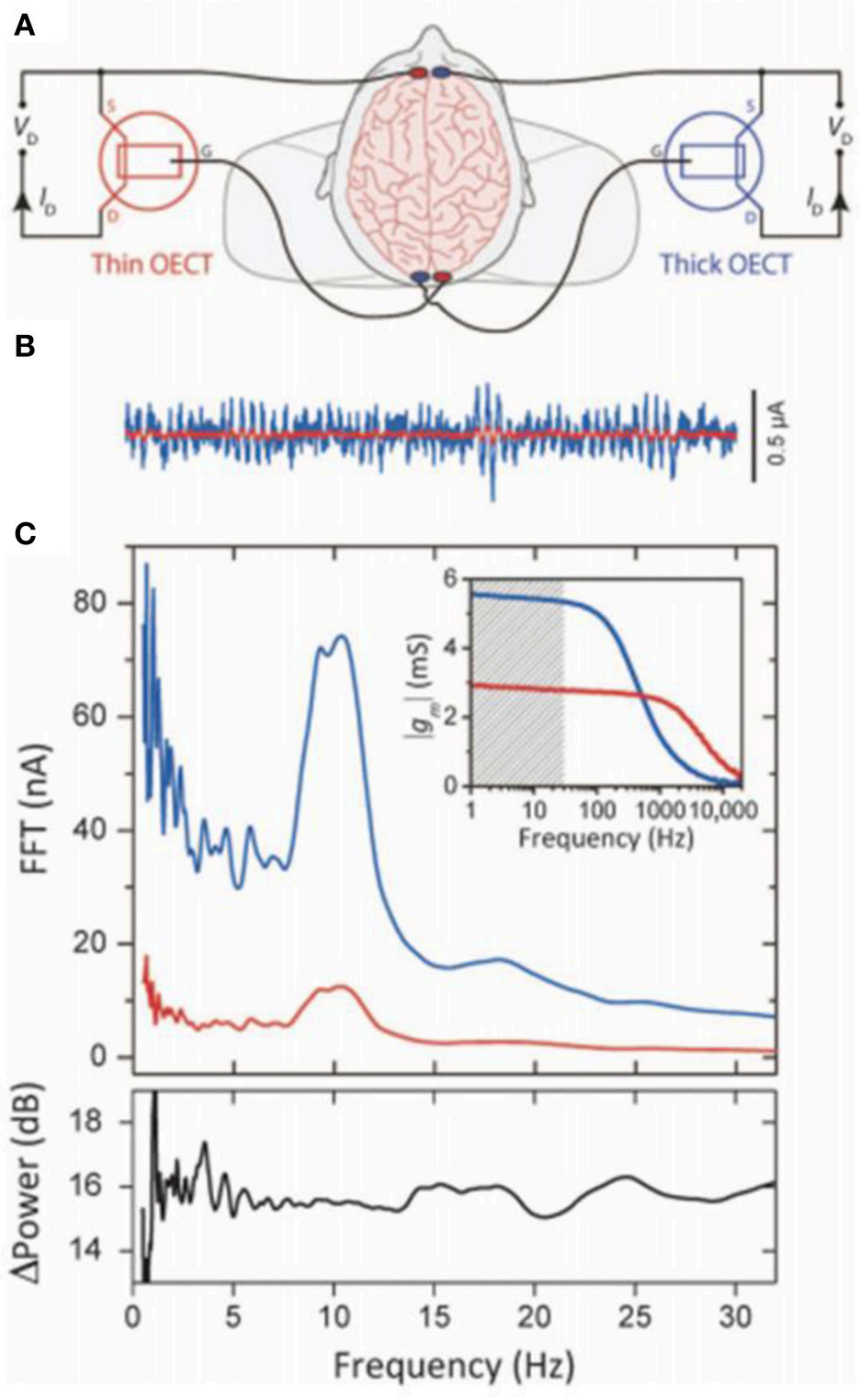
High-transconductance OECTs enhanced EEG recordings. **(A)** Schematic wiring of simultaneously recording human EEG signals by two OECTs. **(B)** The rhythm from a thin (red) and thick (blue) 6-s recordings of OECT. **(C)** Top: fast Fourier transform (FFT) of simultaneous 60 s EEG recordings (inset: response of transconductance frequency, shaded band is the EEG-relevant frequencies). Bottom: the power enhancement recording from the thick compared to the thin device, showing the richer spectral content below the α band and using the thick device leading to the enhanced low-frequency signal (Rivnay et al., 2015a). Reproduced with permission (Rivnay et al., 2015a). Copyright^©^ 2015 American Association for the Advancement of Science.

Implantable ECoG electrodes with flexibility and biocompatibility are of great importance in conforming stable neural interfaces (Campana et al., 2014). Green et al. (2013) examined the long-term stability and injecting limit of PEDOT-coated Pt electrode arrays stimulated under biological electrolytes. The PEDOT-coated Pt electrodes have the charge injection limit of 30 times larger in physiological relevant media and 20 times larger in protein supplemented media compared with bare Pt electrodes. Additionally, the PEDOT-coated electrode that has lower potential excursion can read out signals in the visual cortex in *in-vivo* studies and electrically stimulated potentials. Concerning the continual stimulation, the PEDOT electrodes perform high duration and amplification. The high frequency pulsing of 2,000 Hz stimulation rate, didn't induce loss in stimulation performance, so that the electrodes could be applied to evoke the neural response of injected charge at the average of 76.0 nC (67.0 μC cm^−2^), which has no obviously difference with the Pt electrodes, with the average threshold response of 84.5 nC (74.5 μC cm^−2^). Tunable channel thickness can result in tunable transconductance (Rivnay et al., 2015a), which relatively demonstrate a novel high-performance method for human brain rhythms using the organic transistors (Figure 10).

Information transfer within the brain occurs in the network where neurons are associated with each other by enormous synapses and immersed in the same physiological environments (George et al., 2019). The electrolyte builds complex connections between different synapses, and the global regulations would induce the overall behavior of a large neuro population (Bettinger, 2018). Gkoupidenis et al. (2017) firstly demonstrated the concept of global regulation through the electrolyte gating with neuromorphic devices, they established an array device based on PEDOT: PSS to access the global control characteristics of neurons in a common electrolyte. The array of two-terminal devices immersed in 100 mM electrolyte and the PEDOT: PSS channels of each device serves as the hard connection between the input and output wire. The voltage bias applied on the electrolyte and concentration of ions globally modulate the hard connections. To make use of this effect, they proved synchronism of I/O transmission and the behavior of global clock, similar with the coupling of individual neuron activity and global oscillation in neural networks. These results show the electrolyte gating is advantageous to realize the neuromorphic devices with higher precision and complexity. Khodagholy et al. (2016) introduced a neural interface device (NeuroGrid) (Figure 10) that can simultaneously recording LFP and the action potential from brain surface. The recording signals with the PEDOT: PSS-based NeuroGrids between mid-γ waves (75 to 90 Hz) decreased correctly to the distance on separate electrodes, demonstrating NeuroGrids have high–spatial resolution when representing activities of small populations of neurons.

Coupling of OECTs with *in vivo* electrophysiological recordings is necessary and beneficial for brain-machine interfaces (Won et al., 2018; Gu et al., 2019). Because the organic transistors with conducting channels can connect both electronics and ions within the electrolyte. Ions can penetrate into the volume of the polymer channel and compensate the semiconductor, which will consequently change the conducting ability and thus modulate the current density of the channel. There are many factors can be tuned to obtain the ideal devices, such as channel thickness and the geometry of the channel. van de Burgt et al. (2018), demonstrated the ECoG recording of OECTs. The device, with an organic electrochemical transistor embedded on a thin organic film, recording *in vivo* epileptiform discharge, and performing great signal-to-noise ratio compared with state-of-the-art surface electrode. In addition, the OECTs can record the low-amplitude activities on the surface of the brain, which was superior to the traditional surface electrodes (Martin and Malliaras, 2016). Finally, the biocompatible, mechanical flexible devices for recording brain activities with superior signal-to-noise ratio hold promising future for medical applications.

## Conclusions

Organic bioelectronics, as a promising popular interdisciplinary subject, targets to interface electronics and biology so that improve present biomedical technologies. The OECT lies in the center of this field mainly due to its initial nature of interfacing with biological components and has been proven to exceed general devices such as traditional electrochemical method. Moreover, various biological applications have been explored, including detecting metabolite and monitoring physiological signals since the OECTs bear the following advantages: (i) Stability is an extremely valued performance for biosensing. The OECT has been proven to work stably in various electrolytes, such as cerebrospinal fluid, cell media, sweat, and tears. The durability in these complex electrolytes can last; (ii) Sensitivity could be largely improved by OECT. Since small changes on the input will lead to enormous changes on the output. OECTs display high transconductance values, characteristically high gain, and the time response can be improved by tuning the geometry and size of the channel; (iii) OECTs could be easily integrated with biological system, such as individual cells, tissues, and even whole organs. Moreover, the gate electrode and conducting polymers could be easily modified by various recognition molecules; (iv) Compatibility with photo lithographical techniques also promote the microscale devices construction, especially interesting for monitoring activities of *in vitro* or *in vivo* cells.

In the past decade there has been noticeable progress on the advancing of OECTs for biological applications, however, numerous challenges still remain. These including: (i) The discovery and implementation of innovative active conducting polymers with enhanced properties with regard to conductivity, stability, patterning and restorability; (ii) The fabrication of devices for integrating multiplexed miniaturized arrays on versatile sensors, which may possibly power, record, or transmit the signals; (iii) The selectivity remains a big challenge for multi-chemicals and molecules sensing, especially for complicated samples (e.g., micro dialysate from brain); (iv) The response time was slow for the OECT devices due to the ions of electrolyte moving into or out of the volume of channel. The solution of these questions would largely accelerate the wide application of OECT in biological system, especially for the development of wearable device and brain-machine interface.

## Author Contributions

LB, CE, and WL collected the publications related to this review article, wrote the first draft of the manuscript and made subsequent corrections. PY, JF, and LM completed critical literature analysis and checked subsequent manuscript drafts.

### Conflict of Interest Statement

The authors declare that the research was conducted in the absence of any commercial or financial relationships that could be construed as a potential conflict of interest.
